# Cariogenic potential of the *Streptococcus mutans* Cid/Lrg system: an *in vivo* animal case study

**DOI:** 10.1128/spectrum.01660-24

**Published:** 2024-10-30

**Authors:** Sang-Joon Ahn, Kelly C. Rice, David J. Culp

**Affiliations:** 1 Department of Oral Biology, College of Dentistry, University of Florida, Gainesville, Florida, USA; 2 Department of Microbiology and Cell Science, Institute of Food and Agricultural Sciences, University of Florida, Gainesville, Florida, USA; Michigan State University, East Lansing, Michigan, USA

**Keywords:** *Streptococcus mutans*, oxidative stress, caries, sucrose, *in vivo *mouse model, Lrg/Cid

## Abstract

In our prior study using a dual-species (*Streptococcus mutans/Streptococcus gordonii*) competitive mouse caries model to investigate the contribution of *S. mutans* LrgAB to *in vivo* fitness, *S. mutans* wild-type and Δ*lrgAB* mutants consistently outnumbered *S. gordonii* and had high caries scores, even though the Δ*lrgAB* mutant is highly sensitive to oxidative stress. To determine whether the highly cariogenic sucrose diet used in the previous study masked the contribution of LrgAB to competitive fitness of *S. mutans* against *S. gordonii*, we recapitulated our previous mouse caries experiment with a modification in which 4% sucrose drinking water was replaced with sterile water, hypothesized to decrease the frequency of exposure of mice to sucrose, a determinant in the cariogenicity of *S. mutans*. Given that both *S. mutans* Δ*lrgAB* and Δ*cidB* mutants are sensitive to oxidative stress and share similar transcriptional profiles, these strains, as well as wild-type UA159, were tested in this modified dual-species mouse caries model. When comparing between groups the colonization within molar dental biofilms of *S. mutans* strains, Δ*lrgAB* mutant was at a level similar to the wild type, whereas *S. mutans* Δ*cidB* was modestly lower than both wild-type and *S. mutans* Δ*lrgAB*. The severity of total sulcal caries in both the *∆cidB* and *∆lrgAB* mutant infections was significantly lower than that of wild type. These results demonstrate that the Cid/Lrg system aids in *S. mutans* fitness against *S. gordonii* and caries potential *in vivo*, a phenotype likely masked in our previous study by more frequent exposure to sucrose.

**IMPORTANCE:**

The development of a mature biofilm on the tooth surface is the central event in the pathogenesis of dental caries, which primarily requires that cariogenic organisms withstand the limited resources or environmental fluctuations experienced in the oral cavity. The sensitive and heterogeneous response of the *cid* and *lrg* operons to complex external signals has been hypothesized to trigger differentiation of the *Streptococcus mutans* biofilm community into distinct functional subpopulations to promote survival and persistence of the *S. mutans* community when challenged by an unfavorable environment. The study described herein enlightens our understanding of how Cid/Lrg contributes to *S. mutans* pathogenic potential *in vivo* (caries development), warranting further research regarding the adaptive role of Cid/Lrg system in human oral biofilms toward the development of anti-caries strategies directed at the Cid/Lrg system.

## OBSERVATION

Dental caries (tooth decay) is a complex biofilm disease that develops through the survival and accumulation of cariogenic bacteria, including *Streptococcus mutans*, resulting in tooth demineralization (1–5). The ability of cariogenic bacteria to respond rapidly and efficiently to environmental variables (i.e., pH, carbohydrate source/availability, and oxygen) favorably impacts their ability to form persistent biofilms. Studies of the homologous *lrgAB* and *cidAB* operons revealed that mutation of either *lrgAB* or *cidB* renders *S. mutans* more sensitive to environmental stressors, including oxygen (6–8), and these operons are genetically and metabolically cross-regulated (9–11). Furthermore, *lrgAB* encodes a stationary phase pyruvate uptake system (12), broadly expressed across cell subpopulations with a strongly bimodal character in stationary phase cultures (13). The Cid/Lrg system is thus hypothesized to initiate differentiation of the *S. mutans* biofilm community into functional subpopulations, enabling community-wide survival under extreme environmental challenges (12, 13). Moreover, the *cid* and *lrg* operons affect other comprehensive virulence traits (autolysis, biofilm development, and genetic competence) required for survival in the oral cavity (6–8).

In previous dual-species competition and biofilm models between cariogenic *S. mutans* and H_2_O_2_-generating *Streptococcus gordonii* DL1 *in vitro* and in a mouse caries model (14), we found that *in vitro* outcomes diverged from outcomes *in vivo*. In particular, despite prior dental colonization by DL1, the oxygen/H_2_O_2_-sensitive *S. mutans* Δ*lrgAB* mutant (7, 15) consistently outnumbered DL1, leading to high caries scores (14). This result reinforced the importance of considering how factors such as diet, interactions with oral commensals, and host factors contribute to evaluating the role of specific *S. mutans* system(s) in its competitive fitness *in vivo*. We hypothesized that the highly cariogenic sucrose diet imparted an inherent competitive advantage to the *S. mutans* Δ*lrgAB* mutant. Dietary sucrose triggers *S. mutans* production of extracellular glucans, creating an anaerobic dental biofilm microenvironment considered disadvantageous to the oxygen-dependent generation of competitive H_2_O_2_ by *S. gordonii*. Additionally, *S. mutans* produces acids from dietary sucrose, lowering biofilm pH and leading to higher proportions of other acidogenic and aciduric species (2, 16–18). Accordingly, we detected comparable amounts of lactate in dental biofilms of mice infected with or without *S. mutans* (14), suggesting commensal fermentation of sucrose-fostered *S. mutans’* colonization.

**FIG 1:**
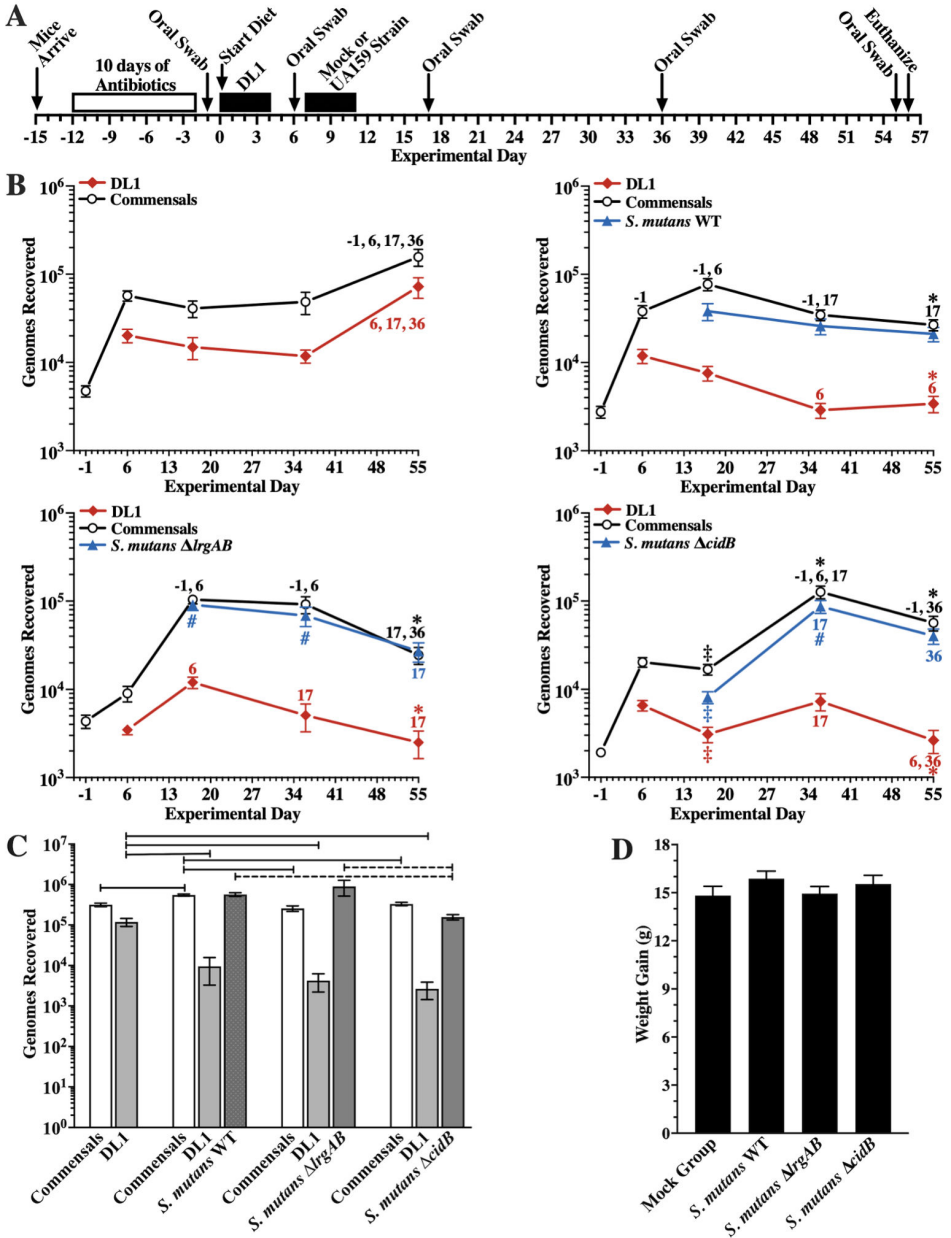
Comparisons of oral and molar colonization of mice by recovered mouse oral commensals and *S. gordonii* DL1, either alone or when in competition with *S. mutans* UA159, *S. mutans* Δ*lrgAB,* or *S. mutans* Δ*cidB*. Also shown are weight gains for each group of mice. (**A**) Timeline of key events in the experiment (performed as previously described in (14)), including periods of daily oral inoculations with *S. gordonii* DL1, followed later by either mock inoculations or a strain of *S. mutans*. (**B**) Oral colonization for each of the indicated inoculated strains and mouse oral commensals within each group of 20 mice as the experimental day indicated in (A). Total recovered bacterial genomes and recovered genomes of each inoculated strain in each DNA sample from oral swabs were assessed by qPCR as described in our previous experiment (14). All assays were performed in triplicate, and the results shown are the mean ± standard error for the 20 mice in each group. Statistical comparisons of colonization between groups were by one-way ANOVA with Tukey’s multiple-comparisons test using GraphPad Prism v10.3.0. Comparisons of the results are presented in four ways. First, statistical differences in recoveries (*P* ≤ 0.05) within each group of mice between swabs at different experimental days within an assay (i.e., mouse commensals, *S. gordonii* DL1, or a strain of *S. mutans*). Differences between points on a line are indicated by either the number or numbers of comparably different experimental days shown above or below a point of an assay line of the same color (e.g., −1, 17 above or below a point on a line of the same color indicates the point is statistically different from results at day −1 and day 17). Second, recoveries at each experimental day for mouse commensals and *S. gordonii* DL1 in each of the three groups inoculated with a strain of *S. mutans* are compared with recoveries of the same assay and experimental day for the group that underwent mock inoculations. Statistical differences (*P* ≤ 0.05) in recoveries are indicated by an asterisk (*) above or below the points of a given assay of the same color. Third, recoveries at each experimental day for mouse commensals, *S. gordonii* DL1, or *S. mutans* in the *S. mutans* Δ*lrgAB* and *S. mutans* Δ*cidB* groups are compared with recoveries of the same assay and experimental day for the group inoculated with wild-type *S. mutans* (*S. mutans* WT). Statistical differences (*P* ≤ 0.05) in recoveries are indicated by the pound sign (#) above or below the points of a given assay of the same color. Fourth, recoveries for mouse commensals, *S. gordonii* DL1, or *S. mutans* in the *S. mutans* Δ*lrgAB* group are compared with recoveries of the same assay and experimental day of the *S. mutans* Δ*cidB* group. Statistical differences (*P* ≤ 0.05) in recoveries are indicated by a double dagger (‡) above or below the points of a given assay of the same color for the *S. mutans* Δ*cidB* group. (**C**) Colonization of dental biofilms after sonication of aseptically extracted mandibular molars in ice-cold PBS by the indicated inoculated strains and mouse oral commensals for each group of 20 mice as assessed by qPCR. The ends of solid bars above the two columns indicate statistical differences (*P* ≤ 0.001) in recoveries, whereas the dashed bar indicates *P* < 0.05 by one-way ANOVA with Tukey’s multiple-comparisons test. (**D**) Average weight gains of mice in each of the four groups from experimental day −14 to 56. In comparing all groups against each other, *P* ≥ 0.426 by one-way ANOVA with Tukey’s multiple comparisons test.

Therefore, we performed a follow-up competitive experiment designed to reduce sucrose-dependent competitiveness of *S. mutans* against *S. gordonii* DL1 by replacing sterile sucrose water (4%, wt/vol) with sterile water. In a pilot mouse caries study using powdered Diet 2000 (56% sucrose), we found that substituting sterile water for 5% sucrose water decreased total smooth surface caries, total sulcal caries, and recovery of *S. mutans* from dental biofilms by 84, 62, and 49%, respectively (unpublished observations). The cariogenicity of *S. mutans* is directly related to its frequency of exposure to sucrose (19, 20), suggesting that omitting sucrose from drinking water may directly decrease the incidence of *S. mutans* exposure to sucrose or indirectly promote erosion of powdered sucrose impacted in sulcal spaces and/or dilute unbound sucrose in dental biofilms. Also, we shortened the washout period following antibiotic suppression of oral commensals to presumably enhance DL1 colonization. Furthermore, because of potential functional linkage between Lrg and Cid (9–11), the previously used *lrgAB*-overexpressing strain, SAB161 (14), was replaced by *S. mutans* Δ*cidB*, a strain also oxygen/H_2_O_2_-sensitive with a transcriptomic profile very similar to the Δ*lrgAB* mutant (8, 10). All other aspects of the experimental protocol (Fig. 1A) were identical to our previous experiment ((14); IACUC# 201810470). Briefly, four groups of 20 mice were inoculated with a single pure culture of DL1. One week later, mice underwent either mock inoculations or were inoculated with *S. mutans* wild type, Δ*lrgAB* or Δ*cidB*. Using qPCR assays (14), we estimated oral colonization of each inoculated strain and mouse commensals from oral swabs taken at progressively increasing days, and finally from dental biofilms extracted from isolated mandibular molars (Fig. 1A).

As shown in Fig. 1B, oral colonization of both DL1 and mouse commensals in the mock group was relatively stable throughout the experiment, except for a modest increase by the end, consistent with previous findings using 4% sucrose water (14). In mice subsequently inoculated with either of the *S. mutans* strains, oral colonization of *S. mutans* was stable at a level comparable to mouse commensals. However, although wild-type *S. mutans* was stable throughout the experiment, both mutants were more variable but displayed similar final levels to the wild type. Conversely, recoveries of both commensals and DL1 in all three *S*. *mutans* groups tended to decrease and concluded at levels lower statistically than the mock group.

Interestingly, molar colonization by mouse commensals was slightly higher in the *S. mutans* wild-type group compared with the other three groups (Fig. 1C). The persistence of commensals in dental biofilms suggests a selection for more aciduric species and possibly indigenous acidogenic species, given measurable sulcal caries in the mock group. Also, it is unclear how antibiotic treatment modified the relative distribution of different commensal species. Future efforts are warranted to delineate changes in the population of each commensal species in Specific Pathogen Free (SPF) mice. In dental biofilms, DL1 recoveries were significantly lower (about 10- to 50-fold) in all three groups inoculated with an *S. mutans* strain versus the mock group but were not statistically different between the three *S*. *mutans* groups. Recoveries of the Δ*lrgAB* mutant displayed similar levels to the wild type, whereas the Δ*cidB* mutant was modestly lower than the other two strains. However, compared with the wild type, total sulcal caries were significantly reduced in mice inoculated with the *∆cidB* and *∆lrgAB* strains (Table 1). Moreover, the *∆cidB* strain displayed lower severity of sulcal caries compared with the wild-type and the Δ*lrgAB* mutant, consistent with its lower level of recovery from dental biofilms. The mean increase in body weights between the four groups was similar (*P* > 0.05), consistent with no major differences in caloric intake and diet consumption (Fig. 1D).

**TABLE 1:** Comparisons of the development caries and their severities on molar surfaces of mice inoculated with *S. gordonii* either without or with different strains of *S. mutans*^*a*^

	DL1 +mock	Dl1 + UA159	DL1 + *∆lrgAB*	DL1 + *∆cidB*
Smooth surfaces				
Total—E	0.70 (0.18)	8.45 (0.72) a	5.32 (0.66) a, b	6.65 (0.78) a
Total—Ds	0.05 (0.05)	1.60 (0.27) a	1.00 (0.22) a	1.10 (0.29) a
Total—Dm	0.00 (0.00)	0.15 (0.11)	0.11 (0.07)	0.05 (0.05)
Buccal E	0.45 (0.11)	3.80 (0.33) a	2.11 (0.43) a, b	3.25 (0.51) a
Buccal Ds	0.05 (0.05)	0.95 (0.21) a	0.37 (0.16) b	0.30 (0.16) b
Buccal Dm	0.00 (0.00)	0.00 (0.00)	0.00 (0.00)	0.05 (0.05)
Lingual E	0.25 (0.10)	4.15 (0.53) a	2.90 (0.37) a, b	3.10 (0.39) a
Lingual Ds	0.00 (0.00)	0.65 (0.20) a	0.63 (0.14) a	0.80 (0.21) a
Lingual Dm	0.00 (0.00)	0.15 (0.11)	0.11 (0.07)	0.00 (0.00)
Proximal E	0.00 (0.00)	0.50 (0.14) a	0.32 (0.11)	0.30 (0.10)
Proximal Ds	0.00 (0.00)	0.00 (0.00)	0.00 (0.00)	0.00 (0.00)
Proximal Dm	0.00 (0.00)	0.00 (0.00)	0.00 (0.00)	0.00 (0.00)
Sulcal surfaces				
Total—E	0.65 (0.15)	12.35 (0.96) a	7.53 (0.87) a, b	5.10 (0.71) a, b
Total—Ds	0.05 (0.05)	3.65 (0.49) a	2.37 (0.44) a	1.35 (0.34) a, c
Total—Dm	0.00 (0.00)	1.35 (0.28) a	0.53 (0.16) a, b	0.10 (0.07) b

^*a*^ Mice were inoculated with *S. gordonii* DL1 (DL1) followed 1 week later with either mock inoculations (DL1 + mock) or inoculated with *S. mutans* UA159 (DL1 + UA159), *S. mutans* ∆*lrgAB* (DL1 + ∆*lrgAB*) or *S. mutans* ∆*cidB* (DL1 + *∆cidB*). Values are means ± standard error of Larson’s modified Keyes' scores. 20 mice per group. Total smooth surface caries is the sum of buccal, lingual, and proximal caries. E (enamel affected), Ds (dentin exposed), and Dm (3/4 of the dentin affected). Comparisons by ANOVA with Tukey’s honest significant difference (HSD) post-test. ^a^*P* ≤ 0.05 versus DL1 + mock; ^b^*P* ≤ 0.05 versus DL1 + UA159; ^c^*P* ≤ 0.05 versus DL1 + *∆lrgAB*.

Overall, these results suggest the *∆cidB* mutant was less competitive *in vivo* against either DL1 and/or mouse commensals. Interestingly, induction of *lrg* expression was shown previously to be diminished in the *∆cidB* mutant at the late exponential phase while *cid* expression was unaffected in the *∆lrgAB* mutant (10). Decreased *lrg* expression by the *∆cidB* mutant *in vivo* may therefore impact traits contributing to lower virulence and/or competitive fitness. However, the formation of sucrose-dependent biofilms and expression of glucan-binding proteins GtfB and GtfC, responsible for cell adherence of *S. mutans* to its extracellular glucan matrix (7, 14), is unimpaired by deletion of either *lrgAB* or *cidB* (7). Both mutations are also without effect on autolysis, considered to enhance the survival of the cell population (7). Furthermore, the growth of both mutants is inhibited under aerobic but not anaerobic conditions (7), consistent with diminished oxidative stress tolerance. Further studies are thus warranted to elucidate traits responsible for lower virulence and recoveries of the *∆cidB* mutant compared with the wild-type and *∆lrgAB* mutant, as is further investigation of decreased virulence of the *∆lrgAB* mutant *in vivo*. Results nevertheless suggest Cid/Lrg promotes the cariogenic potential of *S. mutans in vivo* and demonstrate the importance of dietary factors (e.g., sucrose) in the design of animal caries models.
